# The double-sided effects of *Mycobacterium Bovis* bacillus Calmette–Guérin vaccine

**DOI:** 10.1038/s41541-020-00278-0

**Published:** 2021-01-25

**Authors:** Junli Li, Lingjun Zhan, Chuan Qin

**Affiliations:** 1 NHC Key Laboratory of Human Disease Comparative Medicine, Institute of Laboratory Animal Sciences, Chinese Academy of Medical Sciences (CAMS) and Peking Union Medical College (PUMC), Beijing, 100021 P.R. China; 2 Beijing Key Laboratory for Animal Models of Emerging and Reemerging Infectious, Beijing, 100021 P.R. China; 3 Beijing Engineering Research Center for Experimental Animal Models of Human Critical Diseases, Beijing, 100021 P.R. China; 4 Tuberculosis Center, Chinese Academy of Medical Sciences (CAMS), Beijing, 100021 P.R. China

**Keywords:** Bacteria, Vaccines, Vaccines

## Abstract

Bacillus Calmette–Guérin (BCG), the only vaccine proven to be effective against tuberculosis (TB), is the most commonly used vaccine globally. In addition to its effects on mycobacterial diseases, an increasing amount of epidemiological and experimental evidence accumulated since its introduction in 1921 has shown that BCG also exerts non-specific effects against a number of diseases, such as non-mycobacterial infections, allergies and certain malignancies. Recent Corona Virus Disease 2019 (COVID-19) outbreak has put BCG, a classic vaccine with significant non-specific protection, into the spotlight again. This literature review briefly covers the diverse facets of BCG vaccine, providing new perspectives in terms of specific and non-specific protection mechanisms of this old, multifaceted, and controversial vaccine.

## Introduction

Bacillus Calmette–Guérin (BCG), a live-attenuated bacterial vaccine derived from *Mycobacterium bovis* was originally isolated in 1902 from a cow with tuberculosis (TB)^1^. The isolate was cultured continuously for >230 generations for 13 years (1908–1921) to generate a mutant strain with weakened virulence but with high immunogenicity^2^. First used in humans in 1921, BCG vaccine has been included in the infant immunization programs by the World Health Organization (WHO) since 1974. As of 2018, BCG has been used within the national vaccination program of 180 countries or territories in Asia, Africa, Europe, and America, with a coverage range of over 90%^3–5^. Since the 1920s, the original BCG strain has been shipped to 20 different international sites, where the vaccine was repeatedly sub-cultured under different conditions. This has given rise to diverse licensed BCG formulations that are distinct in live mycobacteria content and in genetic composition^6–8^. Currently, the most widely used strains for BCG vaccine production globally include French Pasteur strain (Pasteur 1173P2), Denmark 1331 strain (Danish 1331), Brazil strain (BCG Mearou RJ), Russian strain (Moscow-368), Bulgarian substrain (Sofia SL222), and the Japan 172 strain (Tokyo 172-1)^9,10^.

As one of the oldest and most widely used vaccines in the world, BCG has been administered for nearly a century, with more than four billion of BCG-vaccinated individuals globally^11^. In most countries, BCG is administered to newborns a few hours or days after birth, and it has been shown to exhibit a protective efficacy of 73% and 77%, respectively, against TB meningitis and miliary TB^12–16^. Although BCG was specifically developed as a vaccine for TB, numerous studies have shown that BCG has the ability to induce the so-called Non-Specific Effects (NSEs) that provide effective protection against other infectious diseases. Several epidemiological studies conducted in TB endemic countries have demonstrated that immunization of neonates with BCG can lower neonatal mortality by 50%^17^, which may be attributed to the decreased likelihood of sepsis and respiratory infections observed in children after receiving BCG vaccination^17–20^. Clinical evidence also suggests that BCG may be effective against infections caused by viral pathogens, such as respiratory syncytial virus^21,22^, human papilloma virus^23–25^, and herpes simplex virus^26^. Moreover, an increasing number of animal studies using mouse models have demonstrated the effects of BCG on secondary viral infections. In two separate studies, mice immunized with BCG have been shown to exhibit a significantly lower titer of influenza A virus (H1N1), resulting in a decreased level of inflammation and lung injury, compared with those without BCG immunization^27,28^. Furthermore, other studies have reported that BCG-vaccinated animal models or humans appeared to be more resistant to various viruses, including herpes simplex virus types 1 and 2^29–31^, sendai virus^32^, Japanese encephalitis virus^33^, encephalomyocarditis virus^34,35^, and ectromelia virus^36,37^, or to non-communicable diseases, such as leukemia^38^, allergy^39^, and childhood diabetes^40^.

Remarkably, BCG can be used as an expression vector for recombinant antigens to develop novel vaccines for pathogenic bacteria and viruses^41–45^, as well as for cancer immunotherapy^38,46,47^. Clearly, BCG cannot be regarded as a vaccine with only “Specific Effects” for unilateral prevention of TB. Hence, further understanding on the possible “NSEs” of BCG is required.

## Clinical characterization of BCG vaccination

The safety of BCG has remained as the primary concern regardless of whether it is used for immune prevention or immunotherapy^48^. Adverse reactions in children owing to BCG vaccination have been well reported in countries that routinely administer BCG^49^. Complications arising from BCG vaccination can be either mild or severe^50^. Immune-compromised individuals with conditions such as severe comprehensive immune deficiency, cellular immune deficiency, chronic granulomatous disease, IL-12 and IFN-γ-mediated immune impairment^51^ often show more-severe reactions to BCG vaccine, and should therefore avoid BCG vaccination. Other factors that may contribute to the development of adverse reactions include evaluation criteria employed, potency and dosage of the vaccine strain used, number of immunization applied, route of delivery, age and immune status of the vaccinated individual and the skills of the operator administering the vaccine.

### Normal reactions to BCG vaccination

BCG, as a live bacterial vaccine, can inevitably disseminate beyond the vaccination site and regional lymph nodes to various parts of the body under certain conditions. About 2 weeks after BCG vaccination, an indurated area with an average diameter of ~10 mm typically occurs at the injection site, followed by redness, swelling, suppuration, and spontaneous ulceration, which turns into a crust that falls off on its own after healing in ~6–12 weeks, forming a small permanent scar, commonly known as the “BCG Scar”. It takes about 2 or 3 months after BCG vaccination to develop a “BCG Scar” without any systemic adverse reactions. It is worth noting that a small number of individuals receiving BCG vaccination have been reported to result in slight swelling of the axillary lymph nodes on the same side of the vaccination site^52^. Generally speaking, the reactions described above are normal after BCG vaccination, which are self-limiting without requiring any special medical treatment.

### Adverse reactions to BCG vaccination

BCG is the most widely used vaccine globally with an excellent overall safety record. Adverse reactions and complications after BCG vaccination mainly include mild and transient fever (that alleviates spontaneously after 1–2 days), injection site abscesses^53^ (with a diameter of >10 mm that heal in >12 weeks), lymphadenitis, TB skin rash^54^ (such as scleroderma erythema, scrotal lichen, and TB papules necrosis that occur between 10 days and 2 months after BCG vaccination), osteomyelitis^55,56^, and systemic disseminated BCG infection^57^. It has been reported that ~1 in 2500 recipients of BCG vaccine shows localized BCG-associated mild complications; while one in 100,000 individuals exhibits disseminated severe complications^51,58,59^. As the formation of abscess after vaccination is usually self-limiting and can be resolved without the need for treatment, only a small number of patients require hospitalization and special procedures. BCG lymphadenitis, the most common side effect^49,60–63^ that manifests as local lymph node enlargement can form pustules, ulceration, suppuration, and other clinical abnormalities such as caseous, abscess, and sinus. Statistical analyses have shown that 30%–80% of BCG lymphadenitis can have purulent changes^64^, and that ~15%–30% of non-suppurative lymphadenitis can progress into purulent lymphadenitis^65^. In addition, unexplained change in urine color has been reported as another abnormal reaction after BCG vaccination. Generally, the urine color turns orange on the second day after vaccination and subsequently recovers on its own in ~1 week^65^, hence no intervention is required. It is worth noting that a very small number of BCG recipients may develop allergic purpura, anaphylactic shock, immune thrombocytopenia, and lichenoid skin lesions after vaccination. Although the incidence rate of the complications outlined above has been reported to be low, prompt symptomatic treatment is required, which may include systemic anti-TB treatment if required.

## Specific effects of BCG against TB

### Innate immune protection

**Fig. 1:**
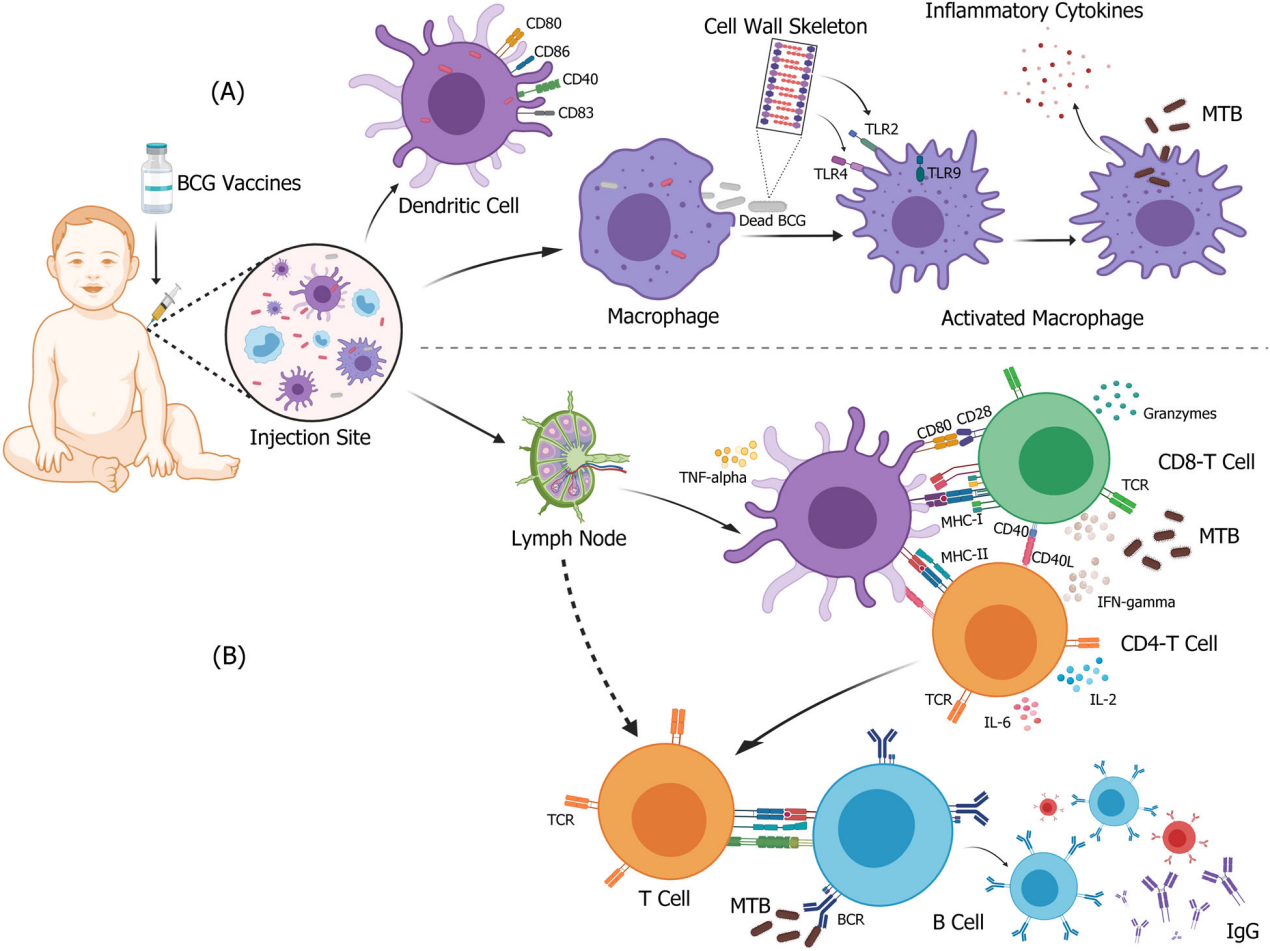
Immune response elicited after BCG vaccination-specific effects for MTB. After BCG vaccine is injected intracutaneously, monocytes, macrophages, and DCs recognize the BCG at the vaccination site and form a strong immune response. **A** BCG is recognized and internalized by DCs or macrophages. The cell wall or other components of BCG act as PRRs that bind to different ligands, upregulate costimulatory molecules of stimulated cells, and activate anti-TB innate immune response. **B** Activated DC cells migrate to lymph nodes to activate mycobacterial specific CD4^+^ and CD8^+^ T cells with a Th1 profile, and subsequently induce the secretion of high levels of cytokines, such as IFN-gamma and granzyme. At the same time, B cells that respond to BCG antigens lead to the production of memory cells, plasma cells, and antigen-specific antibodies. Figure was created using BioRender.

As a complex vaccine consisting live-attenuated mycobacterium, BCG causes local infection and immune activation at the site of administration, where resident monocytes, macrophages, and dendritic cells (DCs) interact with the bacillus^66,67^ (Fig. 1A). BCG internalized by DCs can live up to 2 weeks inside these cells^68^, triggering the upregulation of costimulatory molecules and the production of immune-polarizing cytokines, which are characterized by an increased expression level of CD40, CD80, CD83, and CD86^69,70^. On the other hand, BCG can be phagocytosed and degraded by macrophages, giving rise to immunogenic components of BCG cell wall skeleton, including covalently linked mycolic acid, arabinogalactan, and peptidoglycan^66^ that can strongly stimulate an inflammatory response through the activation of different pattern recognition receptors (PRRs)^69,71–73^. During the recognition of BCG, whilst the signaling of Toll-like receptor (TLR) 2 is triggered via mycobacterial cell wall lipoglycans and lipids, such as lipomannan (LM); the signaling of TLR4 triggered via other mycobacterial proteins, such as multiple heat-shock proteins and glycoproteins^66,74–77^. It has been shown that BCG CpG-DNA can activate intracellular TLR9 in DCs and macrophages, resulting in cell activation and the production of IL-12, TNF-α, and MCP-1, a crucial mediator of Th1 immune response^78–82^. Likewise, complement receptors CR3 and CR4, nucleotide-binding oligomerization domain (NOD)-like receptors and C-type lectins have been shown to interact with bacterial cell wall components and participate in the recognition and internalization of BCG^71,83,84^. Furthermore, studies on BCG-immunized adults have demonstrated reprogramming of monocyte precursors with higher expression of PRRs and greater reactivity to stimuli, such as TLR agonists.

### Adaptive immune protection

Antigen-presenting cells such as DCs, macrophages, and B cells present BCG-derived antigen peptides to major histocompatibility complex (MHC) molecules and primary T cells located in the nearest secondary lymphoid tissue or spleen to initiate an adaptive immune response^85^ (Fig. 1B). Activated DCs at the BCG inoculation site migrate to the draining lymph nodes, and subsequently secrete TNF-α, IL-6, and IL-12 to activate both CD4^+^ and CD8^+^ T cells^86–88^. Activated CD4^+^ and CD8^+^ T cells produce high levels of IFN-γ, which ultimately increases the anti-mycobacterial activity of macrophages^85,89,90^. This explains the increased sensitivity to TB in humans with *IFN-γ* mutations^91^ and in mice with *IFN-γ* gene disrupted^91^. At the same time, the enhancement of antigen-specific T-cell response is co-stimulated by neutrophils and activated DCs^92^. Studies have shown that in newborns, a large number of BCG-specific CD4^+^ and CD8^+^ T cells can be detected in peripheral blood 10 weeks after BCG vaccination. In addition, higher levels of IFN-γ, Granzyme, TNF-α, and IL-2 can be detected in the serum^93–95^. In the development of BCG or TB-related vaccines, the dominant concept of infectious disease immunology lies within the Th1/Th2 paradigm, in which helper T (Th) cells are assigned an exclusive division of labor^96^. According to this paradigm, whilst Th1 cells protect the host from intracellular pathogens (including *Mycobacterium tuberculosis*, MTB), Th2 cells protect the host against extracellular pathogens^96^. Therefore, in the case of TB, IFN-γ secreted by Th1 cells has been used as a criterion for evaluating the protective immunity against BCG. However, there is a lack of knowledge in understanding the BCG-induced immune protection and TB immunology. Therefore, further studies are required to show that IFN-γ is indeed the correct marker of immune protection induced by BCG.

The role of humoral immunity in TB is often neglected^66^. Therefore, the response of B cells to BCG vaccination has also been ignored. However, since recent studies have indicated that antibodies have a role in the protection against TB, there has been a growing research interest in determining their relevance to vaccine development. In fact, potential mechanisms of antibody-mediated immunity against MTB include enhanced phagocytosis, increased phagolysosome formation, bacterial neutralization, enhanced inflammasome activation, and enhanced cytotoxic natural killer (NK) cell activity. For example, the formation of bacterial–antibody complexes has been shown to cause increased processing and presentation of MTB antigens by phagocytes, which in turn increases T-cell activation and enhances cytotoxic responses^97^. It has been shown that B cells can also regulate neutrophilia during BCG vaccination by modulating IL-17 response, and that a vigorously increased level of early neutrophil can impair the initiation of CD4^+^ T cells by affecting the migration of DCs to draining lymph nodes, and eventually trigger Th1 immune response that is not conducive to antibody production^98^. A clinical study on BCG has found that, compared with that of unvaccinated volunteers, the number of PPD-specific memory B cells in PBMCs of BCG-vaccinated is significantly higher, but they lacked B-cell reactivity to ESAT-6 or CFP-10^99^. It has been shown that B-cell response induced within 4–8 weeks after BCG vaccination can increase the production of IgG and induce long-lived memory B cells^99,100^. An antibody response to Ag85 complex has been found to be associated with superior outcome in a cohort of MTB-infected Mexican Indians^101^. Similarly, low-titer antibodies targeting the surface glycolipid lipid arabinomannan of MTB have been shown to be associated with the distribution of TB in children^102^, indicating that antibody effector functions may play a role in infection control^103^. Moreover, IgA produced due to vaccination from a different route (intranasal vaccination) has been shown to form mucosal immunity that can also prevent TB infection^104^. These antibodies can neutralize and eliminate pathogens, and activate immune responses by regulating the secretion of cytokines, such as TNF-α and IL-1β^105^.

## NSEs mechanism of BCG

Several mechanisms by which BCG provides NSEs protection against respiratory infections have been a subject of active investigation. It has been hypothesized that these non-specific benefits may protect against unrelated infections. The first mechanism, “heterologous T-cell immunity” is mediated by heterologous T-cell memory responses to provide cross-protection^106^. The second mechanism, “trained immunity” relies on reprogramming of innate immune cells that confer non-specific immune memory to innate immune responses^107^. In addition, other mechanisms include bystander cells and antibodies/cross-reactive TCR.

### Heterologous T-cell immunity of BCG vaccination

Heterologous T-cell immunity is based on the effects exerted by the adaptive immune system, where primarily T cells mediate the cross-reactivity between vaccine-related and vaccine-unrelated antigens, giving rise to NSEs in vaccines. As such, T-cell vaccine responses may be impacted or modified through exposures to previously infected pathogens^106,108,109^. Although antigen cross-reactivity may provide some beneficial protective immunity in some cases, the reaction may cause severe immunopathological damage in other cases. Therefore, T-cell-mediated heterologous immunity provides a possible biological mechanism that can affect the immune response in subsequent unrelated infections. This also explains how a vaccine can adversely affect the outcome of secondary infections. The molecular similarity between BCG antigens and viral antigens, such as epitopes, can induce the production of a population of memory B and T cells that recognize both BCG and other pathogens. For example, BCG vaccination can protect mice from vaccinia virus infection by increasing the IFN-γ produced by CD4^+^ cells^110^. In human healthy volunteers, the enhancement of non-specific Th1 and Th17 responses can still be observed within 1 year after BCG vaccination^111^. Similarly, in animal experiments, non-targeted antigens have the effect of activating specific CD4^+^ and CD8^+^ memory cells, thereby regulating the response of Th1 and Th17 to non-mycobacterial secondary infections. In addition, BCG has been shown to cause antigen-independent activation of bystander CD4 and CD8 memory cells^110,112–114^. However, this mechanism is unlikely to explain the diversified protection against different pathogens through BCG vaccination. More importantly, although heterologous Th1/Th17 immunity may mediate the long-term effects of heterologous immunity, it takes at least a couple of weeks to be developed, hence highly unlikely to be responsible for the immediate effects observed in perinatal immunity^18,106^.

### Trained immunity of BCG vaccination

Many organisms lacking adaptive immunity, such as plants^115^ and insects^116,117^ manifest robust immune memory after being exposed to infections. Similar adaptive features of innate immunity have also been demonstrated in mice devoid of functional adaptive immune responses^118,119^. Trained immunity is non-specific innate immune protection, formed by monocytes/macrophages and NK cells of the innate immune system, which responds more rapidly and strongly against secondary infections of different microorganisms, depending on the nature and concentration of the ligand^108^, without involving T and B cells^26,107,120,121^. Originally identified in NK cells, trained immunity has been shown to be BCG-inducible in monocytes.

**Fig. 2:**
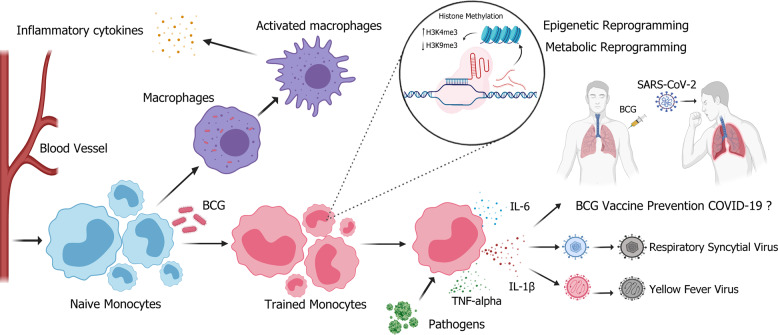
BCG vaccination induces trained immunity. BCG vaccination induces trained immunity of monocytes/macrophages, leading to increased epigenetic reprogramming and host defense capabilities. Chromatin rearrangement induces a “trained” state in monocytes, hence enhancing the effectiveness of innate immune response upon exposure to non-specific pathogens (respiratory syncytial virus or yellow fever virus), and inducing the secretion of proinflammatory cytokines, such as IL-6, IL-1β, and TNF-alpha. However, whether this “training process” can prevent severe acute respiratory syndrome coronavirus 2 (SARS-CoV-2) needs further validation. H3K4me3: trimethylation of lysine at position 4 on histone 3; H3K9me3: trimethylation of lysine at position 9 on histone 3. Figure was created using BioRender.

Epigenetic reprogramming of monocytes in infection or immune sites has been shown as one of the molecular mechanisms that induces trained immunity (Fig. 2). These monocytes undergo histone modifications (such as methylation, acetylation, deamination, and proline isomerization) at the promoter sites of genes encoding inflammatory cytokines, resulting in their ability to respond to new stimuli continuously, leading to an increasingly active immune response upon reactivation. As histone modification is highly dynamic and can be changed within minutes, reprogrammed monocytes can act very rapidly to confer resistance against other pathogens. Epigenetic modification in the immune system involves cell differentiation, inflammation and autoimmune responses^108,122–125^. In supporting the occurrence of trained immunity in the context of BCG immunization, it has been observed that monocytes from adults with BCG vaccination, compared with those without BCG vaccination exhibit increased expression of various surface markers related to immune activation, and produce higher quantities of cytokines, such as IL-1β, IL-6. IFN-γ, and TNF-α upon exposure to infection of various pathogens. In addition, peripheral blood mononuclear cells (PBMCs) have been shown to exhibit an increased histone modification of H3K4me3 and a decreased histone modification of H3K9me3, both associated with the promoters of proinflammatory cytokines TNF-α, IL-6, and TLR4 for transcription activation^126,127^.

Metabolite reprogramming is another molecular mechanism that induces trained immunity (Fig. 2). Some studies have found that, after BCG inoculation, the level of glycolysis metabolism is increased in human monocytes, shifting the cellular metabolic process from oxidative phosphorylation to aerobic glycolysis. Through dectin-1 receptor, β-glucan detected by monocytes/macrophages can activate the PI3K (phosphoinositide 3 kinase)-AKT-mTOR (mammalian target of rapamycin)-HIF-1α (hypoxia-inducible factor-1α) pathway, which subsequently induces a metabolic shift toward aerobic glycolysis; increases glutaminolysis that replenishes the tricarboxylic acid cycle; activates the cholesterol synthesis pathway; and blocks the itaconate pathway^119,128–132^. Consequently, metabolites such as fumarate, succinate, and mevalonate accumulate and then become the cofactors of epigenetic modifiers and act as amplifiers of trained immunity^130,133^. BCG vaccine has been shown to induce genome-wide epigenetic changes, including the monomethylation and trimethylation of histone (H) 3 lysine (K) 4/9, and the acetylation of H3K27 at promoters and enhancers of genes associated with metabolic, immune and host defense pathways^119,127,134^. Hence, trained monocytes/macrophages produce increased levels of cytokines, such as TNF-α, IL-1β, and IL-6, when challenged with microbial constituents^119,127,130,135^.

## NSEs of BCG against infectious diseases other than TB

BCG may also reduce the acquisition of non-mycobacterial infections and decrease the risk of heterologous infections arising from various pathogens, including bacteria, viruses, and malarial parasites. Different clinical studies have been carried out based on the NSEs of BCG (Table 1). However, it is worth noting that besides the causative pathogens, there are many factors can potentially contribute to the heterologous effects of BCG vaccination. Some studies have suggested that diphtheria-tetanus-pertussis vaccine may affect the impact of BCG on childhood mortality, implicating that other vaccines may modulate the NSEs of BCG immunization. In addition, patient age, gender and time post-BCG vaccination may also be the contributing factors of NSEs. The manisfestation of heterologous effects of BCG may be the most apparent for neonates vaccinated at birth. Similarly, studies have shown that in young calves, the effect of BCG-induced innate training in circulating immune populations can last three months^136^. The gradual waning of BCG heterologous protective effect can be explained by the fact that as infants grow older, they become exposed to pathogens more frequently, with the resulting development of the classical immunity against infectious diseases eventually overcoming the heterologous beneficial effects of BCG.

**Table 1:** Clinical trials of BCG as a mitigation factor against infectious diseases other than TB.

Pathogens	ID	Possible mechanism	Phase	Country	Time	Enrollment	Subjects	Aims	Outcome measures
Influenza virus	NCT02114255	Trained immunity	II/III	Netherlands	May 2014–Sep. 2014	40	Healthy adult (male)	To investigate whether prior BCG vaccination improves the efficacy of influenza vaccination in young and/or old healthy volunteers.	✧ Thrombocyte function. ✧ Seroprotection (Influenza antibody titer ≥1:40 or ≥4-fold rise). ✧ Granzyme B, IFN-γ, IL-10, IL-17, and IL-22 by leukocytes ex vivo stimulated with inactivated/live influenza virus. ✧ TNF-α, IL-1β, IFN-γ, IL-10, IL-17, IL-22 by leukocytes ex vivo stimulated with different not-related stimuli.
Hepatitis B	NCT02444611	Trained immunity	NA	Australia	Mar. 2015–Jun. 2016	185	Child	To investigate the effects of BCG and Hepatitis B vaccine, given at birth, on the neonatal immune responses against non-specific antigens.	✧ Cytokine concentrations in response to in vitro stimulation with a range of homologous or heterologous antigens (IL-1α, IL-1β, MCP-1, MIP-1α, MIP-1β, IFN-γ, IL-10, MIF, MIG, TNF-α).
HIV	NCT00331474	Immune activation	I/II	South Africa	May 2006–Aug. 2009	180	Infant	To investigate whether BCG can trigger immune responses against HIV, and whether BCG can regulate the spreading of HIV and early progression to AIDS in babies borned from HIV-positive mothers.	✧ BCG-induced cellular immune responses. ✧ Serum antibody responses. ✧ BCG scarring and TB incidence.
	NCT02062580	Immune activation	II	South Africa	Jun. 2010–Apr. 2012	149	Infant	To investigate whether routine BCG immunization of neonates contributes to generalized immune activation in HIV-exposed infants and increased rates of disease progression in HIV-infected infants.	✧ Percentage of all CD4^+^ T cells expressing HLA-DR. ✧ Percent of CD4^+^ T cells expressing Ki-67 after stimulation in vitro with BCG.
	NCT02606526	Trained immunity	III	Uganda	Jul. 2016–	2200	Infant	To investigate whether BCG vaccination at birth, in a high-risk HIV-exposed can protect infants against serious infections other than TB.	✧ Proportion of infants with severe illness. ✧ Production of TNF-α, IL-1β, IL-6 and IFN-γ in response to mycobacterial and non-mycobacterial antigens. ✧ Adverse events and infant death.
Malarial parasites	NCT00126217	Trained immunity	IV	Guinea	Jul. 2002–Sep. 2006	2871	Child	To investigate whether BCG boosting or no boosting have has an effect on the prevalence of malaria parasitaemia.	✧ Adverse effects. ✧ Mortality till 5 years of age and malaria morbidity/parasitaemia within 12 months after intervention. ✧ Antibody and cellular immune responses 18 months after intervention.
	NCT00131794	Trained immunity	III	Guinea	Jan. 2003–Dec. 2003	1200	Child	Effect of BCG vaccine on morbidity caused by malaria infection.	✧ Incidence of clinical malaria. ✧ Prevalence of malaria parasitemia.
	NCT02692963	Trained immunity	II	Netherlands	Apr. 2016–Feb. 2017	20	Healthy adult	To investigate whether BCG vaccination can offer protection against malaria in the Controlled Human Malaria Infection (CHMI) model.	✧ Frequency and magnitude of adverse events. ✧ Time to blood stage parasitemia detectable by qPCR. ✧ Changes in cellular (innate and adaptive) immune responses. ✧ Changes in plasma cytokine levels.
Bordetella pertussis	NCT02771782	Trained immunity	IV	Netherlands	Jan. 2015–Jul. 2016	75	Healthy adult (female)	To investigate whether BCG vaccination modulates an immune response against non-vaccine target antigens.	✧ Antibody titers. ✧ PBMC cytokine response to homologous or heterologous antigens (IL-6, TNF-α, IL-1β, IL-10, IL-17, IL-22, IFN-γ).
Corynebacterium diphtheria	NCT02771782	Trained immunity	IV	Netherlands	Jan. 2015–Jul. 2016	75	Healthy adult (female)	To investigate whether BCG vaccination modulates an immune response against non-vaccine target antigens.	✧ Antibody titers. ✧ T-cell response and B-cell phenotype analysis. ✧ PBMC cytokine response to homologous or heterologous antigens (IL-6, TNF-α, IL-1β, IL-10, IL-17, IL-22, IFN-γ).

*ID* ClinicalTrials.gov Identifier, *NA* not available, *HLA-DR* human leukocyte antigen DR, *PBMC* peripheral blood mononuclear cell.

## Can BCG confer protection against COVID-19?

The Corona Virus Disease 2019 (COVID-19) pandemic has prompted an urgent need for novel vaccination or interventions to lower the disease morbidity and mortality globally. Promising new trials aiming to ascertain whether this commonly used “old vaccine” is effective against “new disease” COVID-19 have been conducted. BCG has been well documented in the infectious disease literature for its ability to induce NSEs against unrelated conditions. Randomized controlled trials are required to provide direct evidence in order to demonstrate whether BCG vaccination, through trained immunity, is capable of providing protection against COVID-19 (Fig. 2). In vivo studies have shown that BCG can successfully alleviate yellow fever viremia through epigenetic reprogramming of monocytes/macrophages in the human innate immune system^127^. Although BCG has been shown to induce a trained immune response against H7N9 in mouse models, subsequent evaluations show that the immune response is not significantly associated with clinical survival, clinical scores or pulmonary inflammation^137^. The effectiveness of the NSEs of BCG is based on the knowledge that in BCG-vaccinated healthy individuals, innate antibacterial mechanism can be enhanced by the well-trained immune system, which may contribute to the suppression of virus replication and the decrement of viral load, and subsequently result in the alleviation of inflammation and symptoms. However, it is unclear whether older individuals are able to maintain a pool of trained monocytes many years after receiving BCG vaccination. Similarly, in some high-risk individuals, initial defective antiviral response may lead to an increased viral load that stimulates ineffective systemic inflammation and causes serious illness.

There is currently a large amount of literature that review the possibility of utilizing BCG to prevent COVID-19 based on the epidemiological analysis of BCG vaccination policy and the incidence of COVID-19 in different countries. Epidemiological evidence indicates that countries with national universal BCG vaccination programs for TB prevention, compared with those without such programs, have a much lower incidence rate of severe COVID-19 and mortality. However, it is undeniable that different countries have differences in COVID-19 detection capabilities, treatment efficacies and isolation policies, as well as in economy status, demographics, and population genetic structures. It is precisely because of the above-mentioned various constraints that the results of this epidemiological investigation can only be used to explain the possibility of this hypothesis. BCG vaccine has been in short supply owing to high demand and manufacturing restrictions. The global BCG shortage in 2016 and 2019 has led to changes in the management of BCG for the treatment of bladder cancer. Hence, many countries have decreased the dosage and limited the usage of BCG. In order to prevent the over-consumption of BCG vaccine, and to avoid the false impression that BCG vaccine can provide protection against COVID-19, the WHO has recently warned that BCG should not be used indiscriminately in COVID-19 patients until appropriate evidence is available^138^. It is commendable that more than a dozen of new clinical trials of BCG vaccine for COVID-19 have been conducted on healthcare workers and the elderly in countries such as Australia, USA, and Netherlands. These clinical trials may unveil the efficacy of BCG against COVID-19 (Table 2).

**Table 2:** Ongoing clinical trials of BCG as a mitigation factor against COVID-19.

ID	Phase	Country	Time	Enrollment	Subjects	Sponsor	BCG strain
NCT04328441	III	Netherlands	Mar. 2020	1500	Healthy Adult (HCW)	UMC Utrecht	Danish strain 1331
NCT04327206	III	Australia/Spain	Mar. 2020	10078	Healthy Adult (HCW)	Murdoch Childrens Research Institute	Danish strain 1331
NCT04348370	V	USA	Apr. 2020	1800	Healthy Adult (HCW)	Texas A&M University	Tice strain
NCT04417335	V	Netherlands	Apr. 2020	2014	Healthy Adult (≥60 years)	Radboud University	Danish strain 1331
NCT04362124	III	Colombia	Apr. 2020	1000	Healthy Adult (HCW)	Universidad de Antioquia	BCG Liofilizada
NCT04350931	III	Egypt	Apr. 2020	900	Healthy Adult (HCW)	Ain Shams University	Danish Strain 1331
NCT04373291	III	Denmark	May 2020	1500	Healthy Adult (HCW)	University of Southern Denmark Bandim Health Project	Danish strain 1331
NCT04414267	V	Greece	May 2020	900	Healthy Adult (≥50 years)	Hellenic Institute for the Study of Sepsis	Moscow strain 361-1
NCT04379336	III	South Africa	May 2020	500	Healthy Adult (HCW)	TASK Applied Science	Danish strain 1331
NCT04384549	III	France	May 2020	1120	Healthy Adult (HCW)	Assistance Publique - Hôpitaux de Paris	NA
NCT04475302	III	India	Jul. 2020	2175	Healthy Adult (≥60 years)	Tuberculosis Research Center, India	SII strain
NCT04461379	III	Mexico	Jul. 2020	908	Healthy Adult (HCW)	Hospital Universitario	Tokyo 172 strain
NCT04537663	V	Netherlands	Aug. 2020	5200	Healthy Adult (≥60 years)	UMC Utrecht	Danish strain 1331
NCT04369794	V	Brazil	Aug. 2020	1000	Healthy Adult (≥18 years)	University of Campinas	NA
NCT04534803	III	USA	Sep. 2020	2100	Healthy Adult (≥60 years)	Harvard Medical School	Tokyo 172 strain
NCT04542330	III	Denmark	Sep. 2020	1900	Healthy Adult (≥65 years)	Bandim Health Project	Danish strain 1331

*ID* ClinicalTrials.gov Identifier, *HCW* Health Care Workers, *NA* not available, *SII* Serum Institute of India.

## Non-specific immunotherapy using BCG

Besides offering specific prevention against TB or non-specific prevention against other infectious diseases, BCG has also been recognized to exert a non-specific therapeutic effect in patients with non-muscle invasive bladder cancer (NMIBC)^139^. First introduced in 1976 for clinical application in urology, the use of BCG in therapy was subsequently approved by the Food and Drug Administration (FDA) for the treatment of superficial bladder cancer in 1990^140^. Several guidelines, including those outlined by the American Urological Association (AUA)^141^, the International Bladder Cancer Group (IBCG)^142^ and the International Consultation on Urological Diseases (ICUD)^143^ have recommended BCG for NMIBC immunotherapy. BCG is still being considered as one of the standard interventions in alleviating NMIBC progression and recurrence^144,145^. Although BCG has been used for the treatment of NMIBC for nearly 40 years, its therapeutic effect requires further investigation.

**Fig. 3:**
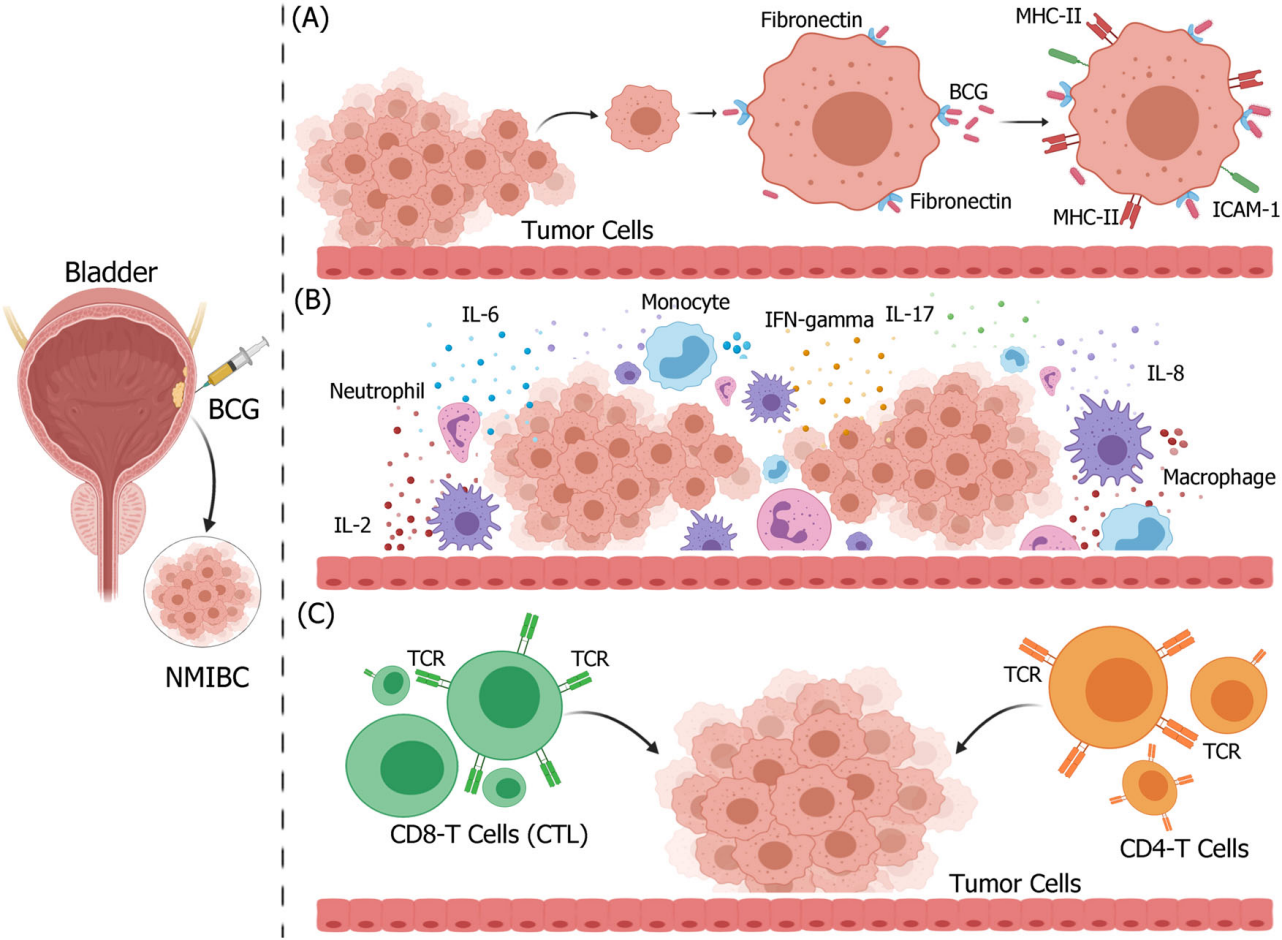
Possible mechanism of BCG non-specific immunotherapy in non-muscle invasive bladder cancer. BCG attached to the urothelium is internalized by bladder cancer cells, causing direct cytotoxicity of immune system effectors to kill bladder cancer cells. **A** BCG attaches to the urothelium via fibronectin and integrin α5β1, and is internalized by bladder cancer cells, owing to oncogenic aberrations that activate macropinocytosis. Following internalization, bladder cancer cells upregulate the expression of MHC-II and ICAM-1. **B** Various immune cells infiltrate bladder tumor tissues, release large amounts of inflammatory cytokines and chemokines, and change the tumor microenvironment. **C** Activated immune cells, such as CD4 and CD8 T cells, are cytotoxic to bladder cancer cells. Figure was created using BioRender.

Intravesical instillation of BCG can induce multiple immune reactions that result in inflammation and subsequent elimination of tumors^146^ (Fig. 3). On one hand, although the vast majority of the original instillation dose of BCG containing several hundred million mycobacteria is washed out from the bladder, the remaining mycobacteria adhere to urothelial and cancer cells through fibronectin, an extracellular glycoprotein distributed in normal and malignant urothelium^147^. To initiate an early immune response, antigen processing of APCs regulated by internalized BCG^148^ enhances the surface expression of MHC-II and intercellular cell adhesion molecule-1 (ICAM-1) of bladder cancer cells^47^. On the other hand, BCG can initiate a cascade of complex inflammatory events within hours of instillation. A marked increase in the number of leukocytes comprising mainly granulocytes, and to a lesser degree, macrophages, and lymphocytes, are detectable in urine^149^. In addition, it has been found that a wide variety of cytokines and chemokines, including Th1 (INF-γ, IL-2, TNF, and IL-12), Th2 (IL-6 and IL-10), IL-8, and IL-17 are released into the urine following BCG therapy, and that the release of cytokines and chemokines is significantly enhanced after BCG re-administration^145,149–163^. More importantly, a large number of neutrophils^163–165^ and monocytes/macrophages have been found to infiltrate the bladder wall, adding additional characteristic cytokines and chemokines to the tumor microenvironment^146,166,167^. Histopathologically, post-treatment bladder biopsies in patients treated with BCG reveal erosion in superficial epithelium, and inflammation in submucosal granulomatous, coupled with edema and noncaseating granulomas surrounded by lymphoplasmacytic and eosinophilic infiltrates^157^. Furthermore, the non-specific anti-tumor effect induced by BCG is considered to be a key intervention for NMIBC immunotherapy. In particular, the anti-tumor effects mediated by CD4^+^ T cells and CD8^+^ cytotoxic T lymphocytes have been well documented. An increased tumor-infiltrating CD4^+^ T-cell count and an increased CD4^+^:CD8^+^ T-cell ratio have been demonstrated to be significantly associated with improved patient response to BCG^156^ in athymic nude mice^168^. Recent studies have also shown that the main cell subsets of leukocytes or neutrophils after repeated BCG instillation produce higher levels of TNF-related apoptosis-inducing ligand (TRAIL) to provide an additional cytotoxic mechanism, which induces apoptotic cell death in TRAIL receptor-expressing tumor cells^79–82,169^.

## Summary and outlook

An increasing number of studies have suggested that BCG vaccination can exert non-specific protective effects to prevent diseases other than TB, and that BCG may be used as a therapeutic agent to modulate the cellular immune response in treating bladder cancer. These findings have important implications for BCG and other vaccines that may exert similar beneficial heterologous effects. Further studies are required to address how BCG regulates T- and B-cell subpopulations with different antigen specificities, as well as how BCG maintains and promotes the proliferation of memory or effector cells. At the same time the role of innate immune response in mediating the NSEs of BCG requires validation. In addition, it will be meaningful to explore whether other immune cell types may participate in non-specific immune protection. In summary, future research findings may improve our understanding on the mechanism of BCG in anti-TB, as well as its NSEs in other infectious diseases.

## References

[1] Daniel TM (2006). The history of tuberculosis. Respir. Med..

[2] Corbel MJ, Fruth U, Griffiths E, Knezevic I (2004). Report on a WHO consultation on the characterisation of BCG strains, Imperial College, London 15-16 December 2003. Vaccine.

[3] WHO. World Health Organization. (2018). Global tuberculosis report 2018.

[4] Ottenhoff TH, Kaufmann SH (2012). Vaccines against tuberculosis: where are we and where do we need to go?. PLoS Pathog..

[5] McShane H (2011). Tuberculosis vaccines: beyond bacille Calmette-Guerin. Philos. Trans. R. Soc. Lond. Ser. B, Biol. Sci..

[6] Brosch R (2007). Genome plasticity of BCG and impact on vaccine efficacy. Proc. Natl. Acad. Sci. USA.

[7] Favorov M (2012). Comparative tuberculosis (TB) prevention effectiveness in children of Bacillus Calmette-Guerin (BCG) vaccines from different sources, Kazakhstan. PloS ONE.

[8] Mostowy S, Tsolaki AG, Small PM, Behr MA (2003). The in vitro evolution of BCG vaccines. Vaccine.

[9] WHO. V3P database (Data 2017). Available at http://www.who.int/immunization/programmes_systems/procurement/v3p/platform/en/. Accessed Nov 2017.

[10] Li J (2020). Tuberculosis vaccine development: from classic to clinical candidates. Eur. J. Clin. Microbiol. Infect. Dis..

[11] Whittaker E, Goldblatt D, McIntyre P, Levy O (2018). Neonatal immunization: rationale, current state, and future prospects. Front. Immunol..

[12] Colditz GA (1995). The efficacy of bacillus Calmette-Guerin vaccination of newborns and infants in the prevention of tuberculosis: meta-analyses of the published literature. Pediatrics.

[13] Miceli I (1988). Evaluation of the effectiveness of BCG vaccination using the case-control method in Buenos Aires, Argentina. Int. J. Epidemiol..

[14] Trunz BB, Fine P, Dye C (2006). Effect of BCG vaccination on childhood tuberculous meningitis and miliary tuberculosis worldwide: a meta-analysis and assessment of cost-effectiveness. Lancet (Lond., Engl.).

[15] Bonifachich E (2006). Protective effect of Bacillus Calmette-Guerin (BCG) vaccination in children with extra-pulmonary tuberculosis, but not the pulmonary disease. A case-control study in Rosario, Argentina. Vaccine.

[16] Roy A (2014). Effect of BCG vaccination against Mycobacterium tuberculosis infection in children: systematic review and meta-analysis. BMJ (Clin. Res. ed.).

[17] Aaby P (2011). Randomized trial of BCG vaccination at birth to low-birth-weight children: beneficial nonspecific effects in the neonatal period?. J. Infect. Dis..

[18] Biering-Sørensen S (2012). Small randomized trial among low-birth-weight children receiving bacillus Calmette-Guérin vaccination at first health center contact. Pediatr. Infect. Dis. J..

[19] Roth A (2004). Low birth weight infants and Calmette-Guérin bacillus vaccination at birth: community study from Guinea-Bissau. Pediatr. Infect. Dis. J..

[20] Biering-Sørensen S (2017). Early BCG-Denmark and neonatal mortality among infants weighing <2500 g: a randomized Controlled Trial. Clin. Infect. Dis..

[21] Stensballe LG (2005). Acute lower respiratory tract infections and respiratory syncytial virus in infants in Guinea-Bissau: a beneficial effect of BCG vaccination for girls community based case-control study. Vaccine.

[22] Wardhana Rifai, Datau EA, Sultana A, Mandang VV, Jim E (2011). The efficacy of Bacillus Calmette-Guerin vaccinations for the prevention of acute upper respiratory tract infection in the elderly. Acta Med. Indonesiana.

[23] Salem A, Nofal A, Hosny D (2013). Treatment of common and plane warts in children with topical viable Bacillus Calmette-Guerin. Pediatr. Dermatol..

[24] Podder I (2017). Immunotherapy in viral warts with intradermal Bacillus Calmette-Guerin vaccine versus intradermal tuberculin purified protein derivative: a double-blind, randomized controlled trial comparing effectiveness and safety in a tertiary care center in Eastern India. Indian J. Dermatol. Venereol. Leprol..

[25] Daulatabad D, Pandhi D, Singal A (2016). BCG vaccine for immunotherapy in warts: is it really safe in a tuberculosis endemic area?. Dermatol. Ther..

[26] Moorlag S, Arts RJW, van Crevel R, Netea MG (2019). Non-specific effects of BCG vaccine on viral infections. Clin. Microbiol. Infect..

[27] Spencer JC, Ganguly R, Waldman RH (1977). Nonspecific protection of mice against influenza virus infection by local or systemic immunization with Bacille Calmette-Guérin. J. Infect. Dis..

[28] Mukherjee S (2017). Boosting efferocytosis in alveolar space using BCG vaccine to protect host against influenza pneumonia. PloS ONE.

[29] Floc’h F, Werner GH (1976). Increased resistance to virus infections of mice inoculated with BCG (Bacillus calmette-guérin). Ann. Immunol..

[30] Starr SE, Visintine AM, Tomeh MO, Nahmias AJ (1976). Effects of immunostimulants on resistance of newborn mice to herpes simplex type 2 infection. Proc. Soc. Exp. Biol. Med..

[31] Ikeda S, Negishi T, Nishimura C (1985). Enhancement of non-specific resistance to viral infection by muramyldipeptide and its analogs. Antivir. Res..

[32] Ishihara C (1987). Suppression of Sendai virus growth by treatment with N alpha-acetylmuramyl-L-alanyl-D-isoglutaminyl-N epsilon-stearoyl-L-lysine in mice. Vaccine.

[33] Kulkarni S (2016). Bacillus Calmette-Guérin confers neuroprotection in a murine model of Japanese Encephalitis. Neuroimmunomodulation.

[34] Lodmell DL, Ewalt LC (1978). Enhanced resistance against encephalomyocarditis virus infection in mice, induced by a nonviable Mycobacterium tuberculosis oil-droplet vaccine. Infect. Immun..

[35] Lodmell DL, Ewalt LC (1978). Induction of enhanced resistance against encephalomyocarditis virus infection of mice by nonviable Mycobacterium tuberculosis: mechanisms of protection. Infect. Immun..

[36] Suenaga T, Okuyama T, Yoshida I, Azuma M (1978). Effect of Mycobacterium tuberculosis BCG infection on the resistance of mice to ectromelia virus infection: participation of interferon in enhanced resistance. Infect. Immun..

[37] Sakuma T, Suenaga T, Yoshida I, Azuma M (1983). Mechanisms of enhanced resistance of Mycobacterium bovis BCG-treated mice to ectromelia virus infection. Infect. Immun..

[38] Morra ME (2017). Early vaccination protects against childhood leukemia: a systematic review and meta-analysis. Sci. Rep..

[39] Thøstesen LM (2018). Neonatal BCG vaccination and atopic dermatitis before 13 months of age: a randomized clinical trial. Allergy.

[40] Rousseau MC, El-Zein M, Conus F, Legault L, Parent ME (2016). Bacillus Calmette-Guérin (BCG) vaccination in infancy and risk of childhood diabetes. Paediatr. Perinat. Epidemiol..

[41] Fennelly GJ, Flynn JL, ter Meulen V, Liebert UG, Bloom BR (1995). Recombinant bacille Calmette-Guérin priming against measles. J. Infect. Dis..

[42] Yu JS (2007). Recombinant Mycobacterium bovis bacillus Calmette-Guerin elicits human immunodeficiency virus type 1 envelope-specific T lymphocytes at mucosal sites. Clin. Vaccine Immunol..

[43] Chapman R, Stutz H, Jacobs W, Shephard E, Williamson AL (2013). Priming with recombinant auxotrophic BCG expressing HIV-1 Gag, RT and Gp120 and boosting with recombinant MVA induces a robust T cell response in mice. PloS ONE.

[44] Palavecino CE, Céspedes PF, Gómez RS, Kalergis AM, Bueno SM (2014). Immunization with a recombinant bacillus Calmette-Guerin strain confers protective Th1 immunity against the human metapneumovirus. J. Immunol..

[45] Soto JA (2018). Recombinant BCG vaccines reduce pneumovirus-caused airway pathology by inducing protective humoral immunity. Front. Immunol..

[46] Pfahlberg A (2002). Inverse association between melanoma and previous vaccinations against tuberculosis and smallpox: results of the FEBIM study. J. invest. Dermatol..

[47] Ikeda N, Toida I, Iwasaki A, Kawai K, Akaza H (2002). Surface antigen expression on bladder tumor cells induced by bacillus Calmette-Guérin (BCG): A role of BCG internalization into tumor cells. Int. J. Urol..

[48] Murphy D, Corner LA, Gormley E (2008). Adverse reactions to Mycobacterium bovis bacille Calmette-Guérin (BCG) vaccination against tuberculosis in humans, veterinary animals and wildlife species. Tuberculosis (Edinb., Scotl.).

[49] Bolger T, O’Connell M, Menon A, Butler K (2006). Complications associated with the bacille Calmette-Guérin vaccination in Ireland. Arch. Dis. Child..

[50] Govindarajan KK, Chai FY (2011). BCG adenitis-need for increased awareness. Malays. J. Med. Sci..

[51] Afshar Paiman S, Siadati A, Mamishi S, Tabatabaie P, Khotaee G (2006). Disseminated Mycobacterium bovis infection after BCG vaccination. Iran. J. Allergy Asthma Immunol..

[52] Barari-Savadkouhi R, Shour A, Masrour-Roudsari J (2016). A study of the incidence of BCG vaccine complications in infants of Babol, Mazandaran (2011-2013). Casp. J. Intern. Med..

[53] Venkataraman A, Yusuff M, Liebeschuetz S, Riddell A, Prendergast AJ (2015). Management and outcome of Bacille Calmette-Guérin vaccine adverse reactions. Vaccine.

[54] Muto J, Kuroda K, Tajima S (2006). Papular tuberculides post-BCG vaccination: case report and review of the literature in Japan. Clin. Exp. Dermatol..

[55] Chan PK, Ng BK, Wong CY (2010). Bacille Calmette-Guérin osteomyelitis of the proximal femur. Hong. Kong Med. J..

[56] Gharehdaghi M, Hassani M, Ghodsi E, Khooei A, Moayedpour A (2015). Bacille Calmette-Guérin osteomyelitis. Arch. Bone Jt. Surg..

[57] Atikan BY, Cavusoglu C, Dortkardesler M, Sozeri B (2016). Assessment of tuberculosis infection during treatment with biologic agents in a BCG-vaccinated pediatric population. Clin. Rheumatol..

[58] Lotte A (1988). Second IUATLD study on complications induced by intradermal BCG-vaccination. Bull. Int. Union Tuberc. Lung Dis..

[59] Marciano BE (2014). BCG vaccination in patients with severe combined immunodeficiency: complications, risks, and vaccination policies. J. Allergy Clin. Immunol..

[60] Mahmoudi S (2015). Adverse reactions to Mycobacterium bovis bacille Calmette-Guérin vaccination against tuberculosis in Iranian children. Clin. Exp. Vaccine Res..

[61] Daei Parizi M, Kardoust Parizi A, Izadipour S (2014). Evaluating clinical course of BCG lymphadenitis and factors affect on it during a 5-year period in Kerman, Iran. J. Trop. Pediatr..

[62] Sharifi Asadi P (2015). Clinical, laboratory and imaging findings of the patients with disseminated bacilli Calmette-Guerin disease. Allergol. Immunopathol..

[63] Bukhari E, Alzahrani M, Alsubaie S, Alrabiaah A, Alzamil F (2012). Bacillus Calmette-Guerin lymphadenitis: a 6-year experience in two Saudi hospitals. Indian J. Pathol. Microbiol..

[64] Kuyucu N, Kuyucu S, Ocal B, Teziç T (1998). Comparison of oral erythromycin, local administration of streptomycin and placebo therapy for nonsuppurative Bacillus Calmette-Guérin lymphadenitis. Pediatr. Infect. Dis. J..

[65] Singla A, Singh S, Goraya JS, Radhika S, Sharma M (2002). The natural course of nonsuppurative Calmette-Guérin bacillus lymphadenitis. Pediatr. Infect. Dis. J..

[66] Dockrell HM, Smith SG (2017). What have we learnt about BCG vaccination in the last 20 years?. Front. Immunol..

[67] Moliva JI, Turner J, Torrelles JB (2017). Immune responses to bacillus Calmette-Guérin vaccination: why do they fail to protect against Mycobacterium tuberculosis?. Front. Immunol..

[68] Jiao X (2002). Dendritic cells are host cells for mycobacteria in vivo that trigger innate and acquired immunity. J. Immunol..

[69] Tsuji S (2000). Maturation of human dendritic cells by cell wall skeleton of Mycobacterium bovis bacillus Calmette-Guérin: involvement of toll-like receptors. Infect. Immun..

[70] Dowling D, Hamilton CM, O’Neill SM (2008). A comparative analysis of cytokine responses, cell surface marker expression and MAPKs in DCs matured with LPS compared with a panel of TLR ligands. Cytokine.

[71] Kleinnijenhuis J, Oosting M, Joosten LA, Netea MG, Van Crevel R (2011). Innate immune recognition of Mycobacterium tuberculosis. Clin. Dev. Immunol..

[72] Azuma I, Ribi EE, Meyer TJ, Zbar B (1974). Biologically active components from mycobacterial cell walls. I. Isolation and composition of cell wall skeleton and component P3. J. Natl. Cancer Inst..

[73] Chatterjee D (1997). The mycobacterial cell wall: structure, biosynthesis and sites of drug action. Curr. Opin. Chem. Biol..

[74] Quesniaux VJ (2004). Toll-like receptor 2 (TLR2)-dependent-positive and TLR2-independent-negative regulation of proinflammatory cytokines by mycobacterial lipomannans. J. Immunol..

[75] Bulut Y (2005). Mycobacterium tuberculosis heat shock proteins use diverse Toll-like receptor pathways to activate pro-inflammatory signals. J. Biol. Chem..

[76] Kim K (2012). Mycobacterium tuberculosis Rv0652 stimulates production of tumour necrosis factor and monocytes chemoattractant protein-1 in macrophages through the Toll-like receptor 4 pathway. Immunology.

[77] Jung SB (2006). The mycobacterial 38-kilodalton glycolipoprotein antigen activates the mitogen-activated protein kinase pathway and release of proinflammatory cytokines through Toll-like receptors 2 and 4 in human monocytes. Infect. Immun..

[78] Li J (2020). Unmethylated CpG motif-containing genomic DNA fragment of Bacillus calmette-guerin promotes macrophage functions through TLR9-mediated activation of NF-κB and MAPKs signaling pathways. Innate Immun..

[79] Tokunaga T, Yamamoto T, Yamamoto S (1999). How BCG led to the discovery of immunostimulatory DNA. Jpn. J. Infect. Dis..

[80] Iwasaki A, Medzhitov R (2004). Toll-like receptor control of the adaptive immune responses. Nat. Immunol..

[81] von Meyenn F (2006). Toll-like receptor 9 contributes to recognition of Mycobacterium bovis Bacillus Calmette-Guérin by Flt3-ligand generated dendritic cells. Immunobiology.

[82] Bafica A (2005). TLR9 regulates Th1 responses and cooperates with TLR2 in mediating optimal resistance to Mycobacterium tuberculosis. J. Exp. Med..

[83] Gagliardi MC (2005). Mycobacterium bovis Bacillus Calmette-Guerin infects DC-SIGN- dendritic cell and causes the inhibition of IL-12 and the enhancement of IL-10 production. J. Leukoc. Biol..

[84] Clarke TB (2010). Recognition of peptidoglycan from the microbiota by Nod1 enhances systemic innate immunity. Nat. Med..

[85] Kaufmann SH (2013). Tuberculosis vaccines: time to think about the next generation. Semin. Immunol..

[86] Bollampalli VP (2015). BCG skin infection triggers IL-1R-MyD88-dependent migration of EpCAMlow CD11bhigh skin dendritic cells to draining lymph node during CD4 + T-cell priming. PLoS Pathog..

[87] Bizzell E (2018). Deletion of BCG Hip1 protease enhances dendritic cell and CD4 T cell responses. J. Leukoc. Biol..

[88] Su H, Peng B, Zhang Z, Liu Z, Zhang Z (2019). The Mycobacterium tuberculosis glycoprotein Rv1016c protein inhibits dendritic cell maturation, and impairs Th1 /Th17 responses during mycobacteria infection. Mol. Immunol..

[89] Bertholet S (2008). Identification of human T cell antigens for the development of vaccines against Mycobacterium tuberculosis. J. Immunol..

[90] Andersen, P. & Kaufmann, S. H. Novel vaccination strategies against tuberculosis. *Cold Spring Harb. Perspect. Med.* **4**. 10.1101/cshperspect.a018523 (2014). 10.1101/cshperspect.a018523 PMC4031959 24890836

[91] Rossouw M, Nel HJ, Cooke GS, van Helden PD, Hoal EG (2003). Association between tuberculosis and a polymorphic NFkappaB binding site in the interferon gamma gene. Lancet (Lond., Engl.).

[92] Morel C (2008). Mycobacterium bovis BCG-infected neutrophils and dendritic cells cooperate to induce specific T cell responses in humans and mice. Eur. J. Immunol..

[93] Hanekom WA (2005). The immune response to BCG vaccination of newborns. Ann. N. Y. Acad. Sci..

[94] Soares AP (2013). Longitudinal changes in CD4( + ) T-cell memory responses induced by BCG vaccination of newborns. J. Infect. Dis..

[95] Murray RA (2006). Bacillus Calmette Guerin vaccination of human newborns induces a specific, functional CD8 + T cell response. J. Immunol..

[96] Mosmann TR, Coffman RL (1989). TH1 and TH2 cells: different patterns of lymphokine secretion lead to different functional properties. Annu. Rev. Immunol..

[97] Li H, Javid B (2018). Antibodies and tuberculosis: finally coming of age?. Nat. Rev. Immunol..

[98] Kozakiewicz L (2013). B cells regulate neutrophilia during Mycobacterium tuberculosis infection and BCG vaccination by modulating the interleukin-17 response. PLoS Pathog..

[99] Sebina I (2012). Long-lived memory B-cell responses following BCG vaccination. PloS ONE.

[100] Chen T (2016). Association of human antibodies to arabinomannan with enhanced mycobacterial opsonophagocytosis and intracellular growth reduction. J. Infect. Dis..

[101] Sánchez-Rodríguez C (2002). An IgG antibody response to the antigen 85 complex is associated with good outcome in Mexican Totonaca Indians with pulmonary tuberculosis. Int. J. Tuberc. Lung Dis..

[102] Costello AM (1992). Does antibody to mycobacterial antigens, including lipoarabinomannan, limit dissemination in childhood tuberculosis?. Trans. R. Soc. Trop. Med. Hyg..

[103] Lu LL (2016). A functional role for antibodies in tuberculosis. Cell.

[104] Uranga, S., Marinova, D., Martin, C. & Aguilo, N. Protective efficacy and pulmonary immune response following subcutaneous and intranasal BCG administration in mice. *J. Vis. Exp.*10.3791/54440 (2016). 10.3791/54440 PMC5092042 27684521

[105] Hansen IS, Baeten DLP, den Dunnen J (2019). The inflammatory function of human IgA. Cell Mol. Life Sci..

[106] Welsh RM, Selin LK (2002). No one is naive: the significance of heterologous T-cell immunity. Nat. Rev. Immunol..

[107] Netea MG, Quintin J, van der Meer JW (2011). Trained immunity: a memory for innate host defense. Cell Host Microbe.

[108] Kleinnijenhuis J (2012). Bacille Calmette-Guerin induces NOD2-dependent nonspecific protection from reinfection via epigenetic reprogramming of monocytes. Proc. Natl. Acad. Sci. USA.

[109] Djuardi Y, Sartono E, Wibowo H, Supali T, Yazdanbakhsh M (2010). A longitudinal study of BCG vaccination in early childhood: the development of innate and adaptive immune responses. PloS ONE.

[110] Mathurin KS, Martens GW, Kornfeld H, Welsh RM (2009). CD4 T-cell-mediated heterologous immunity between mycobacteria and poxviruses. J. Virol..

[111] Kleinnijenhuis J (2014). Long-lasting effects of BCG vaccination on both heterologous Th1/Th17 responses and innate trained immunity. J. Innate Immun..

[112] Berg RE, Cordes CJ, Forman J (2002). Contribution of CD8 + T cells to innate immunity: IFN-gamma secretion induced by IL-12 and IL-18. Eur. J. Immunol..

[113] Berg RE, Crossley E, Murray S, Forman J (2003). Memory CD8 + T cells provide innate immune protection against Listeria monocytogenes in the absence of cognate antigen. J. Exp. Med..

[114] Lertmemongkolchai G, Cai G, Hunter CA, Bancroft GJ (2001). Bystander activation of CD8 + T cells contributes to the rapid production of IFN-gamma in response to bacterial pathogens. J. Immunol..

[115] Conrath U (2006). Systemic acquired resistance. Plant Signal. Behav..

[116] Pham LN, Dionne MS, Shirasu-Hiza M, Schneider DS (2007). A specific primed immune response in Drosophila is dependent on phagocytes. PLoS Pathog..

[117] Rodrigues J, Brayner FA, Alves LC, Dixit R, Barillas-Mury C (2010). Hemocyte differentiation mediates innate immune memory in Anopheles gambiae mosquitoes. Science.

[118] Sun JC, Beilke JN, Lanier LL (2009). Adaptive immune features of natural killer cells. Nature.

[119] Quintin J (2012). Candida albicans infection affords protection against reinfection via functional reprogramming of monocytes. Cell Host Microbe.

[120] Kleinnijenhuis J, van Crevel R, Netea MG (2015). Trained immunity: consequences for the heterologous effects of BCG vaccination. Trans. R. Soc. Tropical Med. Hyg..

[121] Covián C (2019). BCG-induced cross-protection and development of trained immunity: implication for vaccine design. Front. Immunol..

[122] Wen H, Dou Y, Hogaboam CM, Kunkel SL (2008). Epigenetic regulation of dendritic cell-derived interleukin-12 facilitates immunosuppression after a severe innate immune response. Blood.

[123] Foster SL, Medzhitov R (2009). Gene-specific control of the TLR-induced inflammatory response. Clin. Immunol..

[124] Doñas C (2013). Trichostatin A promotes the generation and suppressive functions of regulatory T cells. Clin. Dev. Immunol..

[125] Zhang Q, Cao X (2019). Epigenetic regulation of the innate immune response to infection. Nat. Rev. Immunol..

[126] Arts RJ (2015). Long-term in vitro and in vivo effects of γ-irradiated BCG on innate and adaptive immunity. J. Leukoc. Biol..

[127] Arts RJW (2018). BCG vaccination protects against experimental viral infection in humans through the induction of cytokines associated with trained immunity. Cell Host Microbe.

[128] Ifrim DC (2013). Candida albicans primes TLR cytokine responses through a Dectin-1/Raf-1-mediated pathway. J. Immunol..

[129] Cheng SC (2014). mTOR- and HIF-1α-mediated aerobic glycolysis as metabolic basis for trained immunity. Science.

[130] Arts RJ (2016). Glutaminolysis and fumarate accumulation integrate immunometabolic and epigenetic programs in trained immunity. Cell Metab..

[131] Saz-Leal P (2018). Targeting SHIP-1 in myeloid cells enhances trained immunity and boosts response to infection. Cell Rep..

[132] Domínguez-Andrés J (2019). The itaconate pathway is a central regulatory node linking innate immune tolerance and trained immunity. Cell Metab..

[133] Bekkering S (2018). Metabolic induction of trained immunity through the mevalonate pathway. Cell.

[134] Saeed S (2014). Epigenetic programming of monocyte-to-macrophage differentiation and trained innate immunity. Science.

[135] Kaufmann E (2018). BCG educates hematopoietic stem cells to generate protective innate immunity against tuberculosis. Cell.

[136] Guerra-Maupome M, Vang DX, McGill JL (2019). Aerosol vaccination with Bacille Calmette-Guerin induces a trained innate immune phenotype in calves. PloS ONE.

[137] de Bree LCJ (2018). Bacillus Calmette-Guérin-induced trained immunity is not protective for experimental influenza A/Anhui/1/2013 (H7N9) infection in mice. Front. Immunol..

[138] WHO. Bacille Calmette-Guerin (BCG) vaccination and COVID-19. www.who.int/news-room/commentaries/detail/bacille-calmette-gu%C3%A9rin-(bcg)-vaccination-and-covid-19 (2020).

[139] Nykopp TK, Batista da Costa J, Mannas M, Black PC (2018). Current Clinical Trials in non-muscle invasive bladder cancer. Curr. Urol. Rep..

[140] Fuge O, Vasdev N, Allchorne P, Green JS (2015). Immunotherapy for bladder cancer. Res. Rep. Urol..

[141] Hall MC (2007). Guideline for the management of nonmuscle invasive bladder cancer (stages Ta, T1, and Tis): 2007 update. J. Urol..

[142] Zhang J (2019). Management of non-muscle-invasive bladder cancer: quality of clinical practice guidelines and variations in recommendations. BMC Cancer.

[143] Burger M (2013). ICUD-EAU International Consultation on Bladder Cancer 2012: non-muscle-invasive urothelial carcinoma of the bladder. Eur. Urol..

[144] Kamat AM (2015). Expert consensus document: consensus statement on best practice management regarding the use of intravesical immunotherapy with BCG for bladder cancer. Nat. Rev. Urol..

[145] Babjuk M (2017). EAU guidelines on non-muscle-invasive urothelial carcinoma of the bladder: update 2016. Eur. Urol..

[146] Böhle A, Gerdes J, Ulmer AJ, Hofstetter AG, Flad HD (1990). Effects of local bacillus Calmette-Guerin therapy in patients with bladder carcinoma on immunocompetent cells of the bladder wall. J. Urol..

[147] Kavoussi LR, Brown EJ, Ritchey JK, Ratliff TL (1990). Fibronectin-mediated Calmette-Guerin bacillus attachment to murine bladder mucosa. Requirement for the expression of an antitumor response. J. Clin. Investig..

[148] Maksymowych WP, Kane KP (2000). Bacterial modulation of antigen processing and presentation. Microbes Infect..

[149] De Boer EC (1991). Presence of activated lymphocytes in the urine of patients with superficial bladder cancer after intravesical immunotherapy with bacillus Calmette-Guérin. Cancer Immunol. Immunother..

[150] Prescott S, James K, Hargreave TB, Chisholm GD, Smyth JF (1990). Radio-immunoassay detection of interferon-gamma in urine after intravesical Evans BCG therapy. J. Urol..

[151] Nadler R (2003). Interleukin 10 induced augmentation of delayed-type hypersensitivity (DTH) enhances Mycobacterium bovis bacillus Calmette-Guérin (BCG) mediated antitumour activity. Clin. Exp. Immunol..

[152] de Reijke TM (1993). Cytokine production by the human bladder carcinoma cell line T24 in the presence of bacillus Calmette-Guerin (BCG). Urol. Res..

[153] de Boer EC (1997). Role of interleukin-8 in onset of the immune response in intravesical BCG therapy for superficial bladder cancer. Urol. Res..

[154] De Boer EC (1992). Induction of urinary interleukin-1 (IL-1), IL-2, IL-6, and tumour necrosis factor during intravesical immunotherapy with bacillus Calmette-Guérin in superficial bladder cancer. Cancer Immunol. Immunother..

[155] Esuvaranathan K (1995). Interleukin-6 production by bladder tumors is upregulated by BCG immunotherapy. J. Urol..

[156] Pichler R (2016). Tumor-infiltrating immune cell subpopulations influence the oncologic outcome after intravesical Bacillus Calmette-Guérin therapy in bladder cancer. Oncotarget.

[157] Lage JM, Bauer WC, Kelley DR, Ratliff TL, Catalona WJ (1986). Histological parameters and pitfalls in the interpretation of bladder biopsies in bacillus Calmette-Guerin treatment of superficial bladder cancer. J. Urol..

[158] Alexandroff A, Jackson A, Skibinska A, James K (1996). Production of IL-5, a classical T(H)2 cytokine, following bacillus Calmette guerin immunotherapy of bladder cancer. Int. J. Oncol..

[159] O’Donnell MA (1999). Role of IL-12 in the induction and potentiation of IFN-gamma in response to bacillus Calmette-Guérin. J. Immunol..

[160] Jackson AM (1995). Changes in urinary cytokines and soluble intercellular adhesion molecule-1 (ICAM-1) in bladder cancer patients after bacillus Calmette-Guérin (BCG) immunotherapy. Clin. Exp. Immunol..

[161] Eto M (2005). Importance of urinary interleukin-18 in intravesical immunotherapy with bacillus calmette-guérin for superficial bladder tumors. Urol. Int..

[162] Luo Y, Chen X, O’Donnell MA (2007). Mycobacterium bovis bacillus Calmette-Guérin (BCG) induces human CC- and CXC-chemokines in vitro and in vivo. Clin. Exp. Immunol..

[163] de Boer EC (1991). Leukocytes in the urine after intravesical BCG treatment for superficial bladder cancer. A flow cytofluorometric analysis. Urol. Res..

[164] Ludwig AT (2004). Tumor necrosis factor-related apoptosis-inducing ligand: a novel mechanism for Bacillus Calmette-Guérin-induced antitumor activity. Cancer Res..

[165] Suttmann H (2006). Neutrophil granulocytes are required for effective Bacillus Calmette-Guérin immunotherapy of bladder cancer and orchestrate local immune responses. Cancer Res..

[166] Prescott S, James K, Hargreave TB, Chisholm GD, Smyth JF (1992). Intravesical Evans strain BCG therapy: quantitative immunohistochemical analysis of the immune response within the bladder wall. J. Urol..

[167] Suttmann H, Lehan N, Böhle A, Brandau S (2003). Stimulation of neutrophil granulocytes with Mycobacterium bovis bacillus Calmette-Guérin induces changes in phenotype and gene expression and inhibits spontaneous apoptosis. Infect. Immun..

[168] Ratliff TL, Gillen D, Catalona WJ (1987). Requirement of a thymus dependent immune response for BCG-mediated antitumor activity. J. Urol..

[169] Saint F (2001). Leukocyturia as a predictor of tolerance and efficacy of intravesical BCG maintenance therapy for superficial bladder cancer. Urology.

